# Oxygen consumption rate of *Caenorhabditis elegans* as a high-throughput endpoint of toxicity testing using the Seahorse XF^e^96 Extracellular Flux Analyzer

**DOI:** 10.1038/s41598-020-61054-7

**Published:** 2020-03-06

**Authors:** G. Du Preez, H. Fourie, M. Daneel, H. Miller, S. Höss, C. Ricci, G. Engelbrecht, M. Zouhar, V. Wepener

**Affiliations:** 1 Unit for Environmental Sciences and Management, North-West University, Private Bag X6001, Potchefstroom, 2520 South Africa; 2 Agricultural Research Council – Institute for Tropical and Subtropical Crops, Private Bag X11208, Nelspruit, 1200 South Africa; 3 Human Metabolomics, Faculty of Natural Sciences, North-West University, Potchefstroom, 2520 South Africa; 4 Ecossa, Giselastrasse 6, 82319 Starnberg, Germany; 5 University of Bielefeld, Department of Animal Ecology, Konsequenz 45, 33615 Bielefeld, Germany; 6 Centre of Excellence for Nutrition (CEN), North-West University, Potchefstroom, South Africa; 7 Pediatric Epidemiology, Department of Pediatrics, University Medicine Leipzig, Leipzig, Germany; 8 Czech University of Life Sciences, Faculty of Agrobiology, Food and Natural Resources, Department of Plant Protection, Kamycka 129, 165 21 Prague, Czech Republic

**Keywords:** Caenorhabditis elegans, High-throughput screening, Toxicology

## Abstract

*Caenorhabditis elegans* presents functioning, biologically relevant phenotypes and is frequently used as a bioindicator of toxicity. However, most *C. elegans in vivo* effect-assessment methods are laborious and time consuming. Therefore, we developed a novel method to measure the oxygen consumption rate of *C. elegans* as a sublethal endpoint of toxicity. This protocol was tested by exposing 50 larval stage one *C. elegans* individuals for 48 h (at 20 °C) to different concentrations of two toxicants i.e. benzylcetyldimethylammonium chloride (BAC-C16) and cadmium (Cd). Following exposures, the oxygen consumption rate of the *C. elegans* individuals were measured using the high-throughput functionality of the Seahorse XF^e^96 Extracellular Flux Analyzer. Dose-response curves for BAC-C16 (R^2^ = 0.93; *P* = 0.001) and Cd (R^2^ = 0.98; *P* = 0.001) were created. Furthermore, a strong, positive correlation was evidenced between *C. elegans* oxygen consumption rate and a commonly used, ecologically relevant endpoint of toxicity (growth inhibition) for BAC-C16 (R^2^ = 0.93; *P* = 0.0001) and Cd (R^2^ = 0.91; *P* = 0.0001). The data presented in this study show that *C. elegans* oxygen consumption rate can be used as a promising functional measurement of toxicity.

## Introduction

*Caenorhabditis elegans* Maupas, 1900 has been extensively used to study the toxic effect of pollutants, drugs, and environmental samples^1^. One clear benefit is the exposure of an intact animal with functioning digestive, endocrine, neuromuscular, reproductive, and sensory systems^2^, i.e. phenotypes that are biologically relevant^3^. This species is also small in size, easy to culture, and can even be maintained axenically^2,4^. Furthermore, studies have shown that *C. elegans* bioassays can be used to predict mammalian development at a fraction of the cost of traditional animal testing^2,5^. These qualities and benefits complement *C. elegans* as a model organism for toxicity testing, as well as its use in high-throughput assessment protocols^3^, as has been developed for drugs^6^ and pollutants of environmental concern^7^.

Commonly used toxicity endpoints include feeding, fertility, growth, movement, reproduction, and survival of *C. elegans*^1,8^. Respiration of *C. elegans* has also been used, although infrequently, to study the effect of toxicant exposure^9–11^. This endpoint has been shown to serve as an effective measure of toxicity of especially metal pollution in, among others, microbes^12^, daphnia^13^ and earthworms^14^. Therefore, with the development of state-of-the-art, high-throughput respirometers, such as the Seahorse XF^e^96 Extracellular Flux Analyzer, the relevance and applicability of *C. elegans* oxygen consumption rate (OCR) measurements have greatly improved. This has led to the development of acute response protocols that measure *C. elegans* OCR before and after the injection of pre-loaded compounds, typically oligomycin, FCCP, rotenone and antimycin A. Such compounds facilitate the measurement of mitochondrial respiratory chain functionality in organisms by determining, for example, ATP production, proton leak, maximal respiration, spare respiratory capacity and non-mitochondrial respiration^15–17^.

However, with such short exposure periods and the lack of food, these OCR protocols have limited environmental relevance. Longer exposure periods would allow the measurement of a chronic response, which is facilitated by the short life cycle (approximately 3 days at 20 °C) of *C. elegans*^3,18^. Furthermore, by adding a food source, nematode respiration would be sustained^16^ and toxicant bioaccessibility increased as feeding nematodes will ingest dissolved and bacterial-bound substrates^19^. Therefore, the aim of this research was to develop an environmentally relevant *C. elegans* OCR protocol for sublethal toxicity testing by utilizing the high-throughput capabilities of the Seahorse XF^e^96 Extracellular Flux Analyzer.

## Results and discussion

### Food density

**Figure 1:**
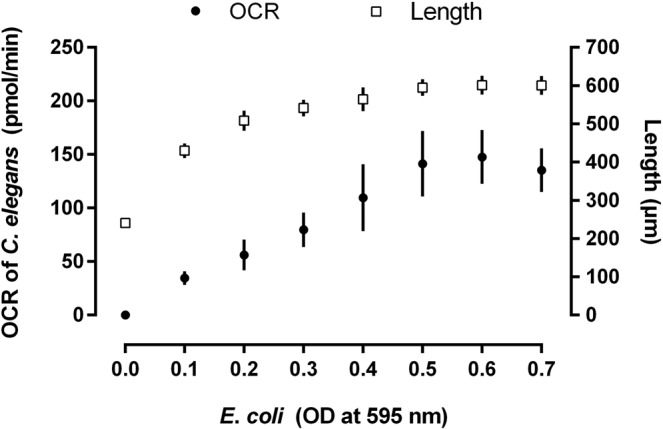
Food density bioassay. Average length and total oxygen consumption rate (OCR) of 50 larval stage one *Caenorhabditis elegans* nematodes are considered at different optical densities (at 595 nm) of its bacterial food source (*Escherichia coli* OP50) after 48 h incubation at 20 °C.

A food density bioassay was performed in order to determine the minimum amount of food (*Escherichia coli* OP50) required to ensure uninhibited nematode development during the present study’s experimental procedures. The relationship between food density and nematode length is illustrated in Fig. 1. Nematode length (or rather growth) was clearly inhibited by decreased food availability, a well-studied response often used to investigate the effect of dietary restrictions on *C. elegans* development^20,21^. Therefore, using a segmented regression model, the minimum density of food that allowed unrestricted nematode development was determined. This model indicated that a plateau for nematode growth was reached at a food (*E. coli*) OD of 0.59 (595 nm). Subsequently, an OD of 0.6 was used for the experimental bioassays.

**Figure 2:**
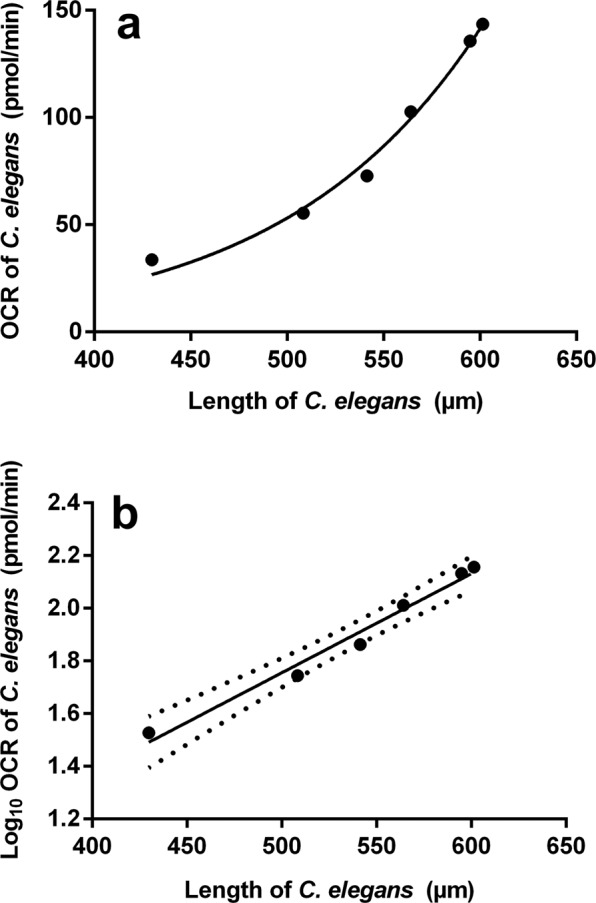
Oxygen consumption rate (OCR) of *Caenorhabditis elegans*. The OCR of *Caenorhabditis elegans* (of 50 larval stage one nematodes) is considered against the average length of such specimens as an (a) exponential (non-linear) growth curve and (b) log_10_ transformed linear model (Y = 0.003761X − 0.1247). The slope of the linear model differed significantly (*P* = 0.0002) from zero with nematode length explaining 98% of the OCR variation.

Furthermore, the relationship between *C. elegans* length and OCR was investigated (Fig. 1) and visualised as a non-linear exponential growth curve (Fig. 2a). In order to infer statistical meaning from this relationship, a linear regression model (Fig. 2b) was applied. The slope of the log_10_ transformed linear model ($Y=0.003761X-0.1247$) differed significantly (*P* = 0.0002) from zero with nematode length explaining 98% of the variation in OCR of *C. elegans*. The 95% confidence bands, as illustrated in Fig. 2b, indicated a low degree of uncertainty. Previous studies have reported on the change in OCR as a function of *C. elegans* larval development and/or adult aging^16,22–26^. However, these observations were mainly made per life stage or for L4 and adult nematodes. It should be noted that the reason for the slight decrease in OCR at a food density of 0.7 OD (at 595 nm) (Fig. 1) remains unknown, however, it is possible, although unconfirmed, that high densities of *E. coli* cells can impair OCR measurement. Nonetheless, the findings presented here clearly show that following incubation, *C. elegans* OCR correlate to its growth stage as influenced by food availability.

### Oxygen consumption rate inhibition due to toxicant exposure

**Figure 3:**
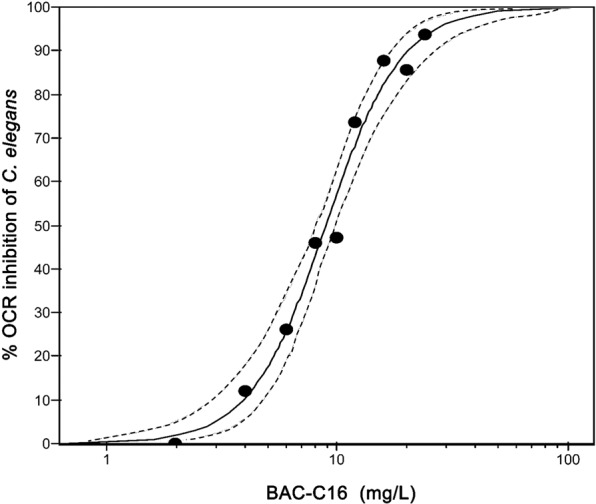
Oxygen consumption rate (OCR) inhibition following benzylcetyldimethylammonium chloride monohydrate (BAC-C16) exposure. Concentration-response curve of *Caenorhabditis elegans* oxygen consumption rate (OCR) inhibition following exposure to BAC-C16. The R^2^ value was calculated as 0.93 (*P* = 0.001) and 95% confidence bands are indicated as dotted lines.

**Figure 4:**
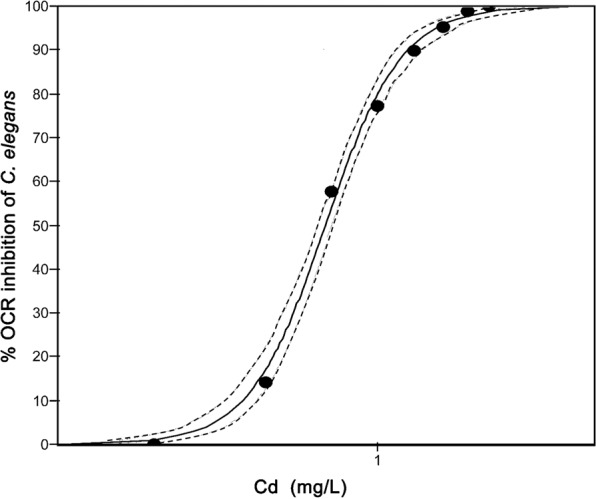
Oxygen consumption rate (OCR) inhibition following cadmium (Cd) exposure. Concentration-response curve of *Caenorhabditis elegans* oxygen consumption rate (OCR) inhibition following exposure to Cd. The R^2^ value was calculated as 0.98 (*P* = 0.001) and 95% confidence bands are indicated as dotted lines.

For each toxicant bioassay the percentage OCR inhibition of *C. elegans* per exposure concentration was measured against a negative control (M9 medium). Following, concentration-response curves for Benzylcetyldimethylammonium chloride monohydrate (BAC-C16) (R^2^ = 0.93; *P* = 0.001) (Fig. 3) and cadmium (Cd) (R^2^ = 0.98; *P* = 0.001) (Fig. 4) were created and used to derive ECx values (Table 1) at the 10, 20, and 50% inhibition levels.

**Table 1:** Effective concentration (EC) values representing 10, 20 and 50% of *Caenorhabditis elegans* oxygen consumption rate (OCR) and growth inhibition.

BAC-C16	OCR			Growth		
	EC10	EC20	EC50	EC10	EC20	EC50
Value (mg/L)	3.99	5.26	8.94	3.4	4.96	9.47
Lower 95% Cl	2.96	4.23	8.07	2.72	4.25	8.8
Upper 95% Cl	4.82	6.08	9.83	4.00	5.58	10.5
**Cadmium**						
Value (mg/L)	0.44	0.52	0.73	0.43	0.56	0.86
Lower 95% Cl	0.39	0.48	0.69	0.32	0.45	0.79
Upper 95% Cl	0.47	0.55	0.76	0.51	0.63	0.94

These values were calculated with a Probit analysis using the linear maximum likelihood regression algorithm. Upper and lower 95% confidence intervals (CI) values, based on Fieller’s Theorem, are also provided.

The BAC-C16 and Cd concentrations at which 50% OCR inhibition of *C. elegans* occurred were calculated as 8.94 mg/L and 0.73 mg/L (Table 1), respectively. Benzylcetyldimethylammonium chloride monohydrate is routinely used as a positive control for *C. elegans* toxicity assays^27,28^, while also serving as the positive control in standardised toxicity testing^29^. Although *C. elegans* OCR inhibition following exposure to BAC-C16 has never been investigated, Schouest *et al*.^11^ used fluorescence oxygen sensing and recorded significant OCR reduction following 24 h exposure of *C. elegans* adults to Cd, as well as other toxicants (e.g. zinc and rotenone). The latter authors calculated an EC50 value (for OCR inhibition) following exposure to Cd of 60.85 µM (6.84 mg/L). This is substantially higher than the EC50 value reported for this study (0.73 mg/L). However, in the present study longer exposure periods of 48 h (vs 24 h) and larval stage one (vs adult) nematodes were used. It is known that longer exposure periods can lead to greater toxicity^30^, while larval stages are typically more sensitive to metal exposure than adults^31^.

Also worth considering is the sensitivity of OCR compared to reproduction, since the latter is regarded as one of the most sensitive *C. elegans* endpoints of toxicity^19,29^. Comparing our results to the findings of other studies, it seems that OCR might be slightly less sensitive than reproduction (EC50 of BAC-C16: 7.5 mg/L; EC50 of Cd: 0.21 mg/L)^19,27^. However, these direct comparisons should be made with caution since different exposure periods were used for deriving ECx values for OCR (48 h) and reproduction (96 h).

### Growth inhibition due to toxicant exposure

**Figure 5:**
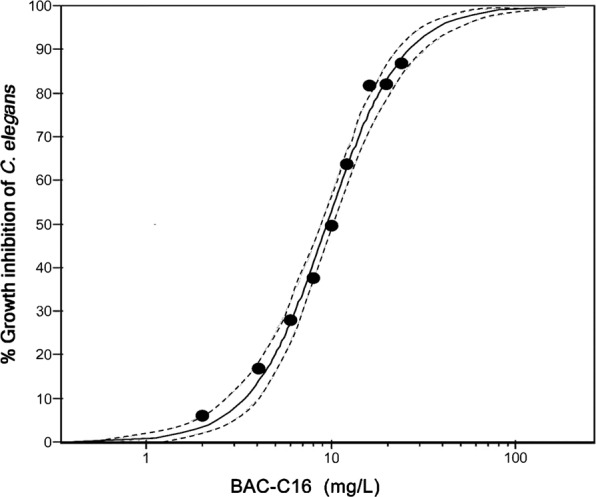
Growth inhibition following benzylcetyldimethylammonium chloride monohydrate (BAC-C16) exposure. Concentration-response curve of *Caenorhabditis elegans* growth inhibition following exposure to BAC-C16. The R^2^ value was calculated as 0.97 (*P* = 0.001) and 95% confidence bands are indicated as dotted lines.

**Figure 6:**
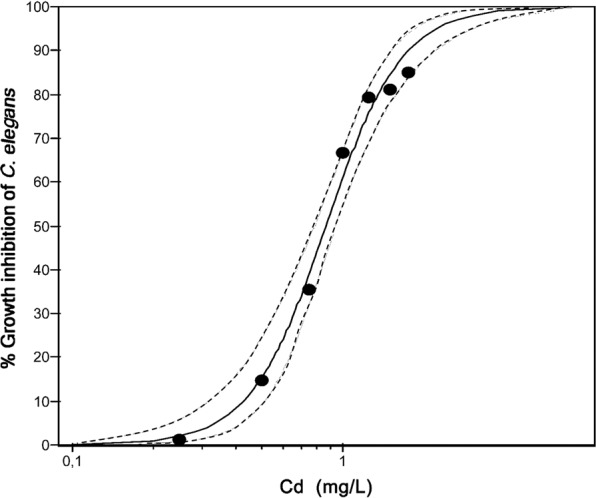
Growth inhibition following cadmium (Cd) exposure. Concentration-response curve of *Caenorhabditis elegans* growth inhibition following exposure to Cd. The R^2^ value was calculated as 0.95 (*P* = 0.001) and 95% confidence bands are indicated as dotted lines.

*Caenorhabditis elegans* growth inhibition was also investigated for two reasons: (1) a strong relationship, as evidenced in the food density bioassay, existed between *C. elegans* OCR and length and (2) *C. elegans* growth inhibition is routinely used as an endpoint of toxicity and can therefore be used to evaluate the sensitivity of *C. elegans* OCR inhibition as an endpoint of toxicity. Growth inhibition concentration-response curves for BAC-C16 (R^2^ = 0.97, *P* = 0.001) and Cd (R^2^ = 0.95, *P* = 0.001) are illustrated on Figs. 5 and 6, respectively.

The BAC-C16 concentration at which 50% growth inhibition of *C. elegans* occurred was calculated as 9.47 mg/L (Table 1). Although EC values of *C. elegans* OCR inhibition for BAC-C16 have not previously been reported, ISO10872^29^ states that the EC50 value for BAC-C16 growth inhibition typically ranges between 8 and 22 mg/L. Most studies have reported EC50 values of approximately 15 mg/L^27,28^, however, Schertzinger *et al*.^32^ reported EC50 values for two separate tests of 9.1 and 10.8 mg/L, respectively. The latter values are therefore similar to results reported in this study.

According to Hanna *et al*.^28^ growth inhibition by BAC-C16 is substantially influenced by food density, with lower densities presenting higher inhibition rates. However, this was not viewed as a concern during the present study as the minimum required amount of food for *C. elegans* was used at a constant density in control and exposure wells of toxicant bioassays.

The Cd concentration at which 50% growth inhibition of *C. elegans* occurred was calculated as 0.86 mg/L (Table 1). As with BAC-C16, Cd toxicity has been linked to food density, with increasing toxicity as food density decreases^19^. The latter authors attributed this to a decrease in the bioavailability of freely dissolved Cd at high bacterial densities. Traunspurger *et al*.^33^ reported a lowest observed effect concentration (LOEC; 72 h exposure) of 0.14 mg/L for the growth of *C. elegans*. This can be compared to the present study’s EC10 and EC20 values of 0.43 and 0.56 mg/L, respectively, following 48 h exposure. Van Kessel *et al*.^34^ on the other hand, showed a substantially higher LOEC (11.2 mg/L) for *C. elegans* after 48 h exposure to Cd. However, this was in the absence of food, which results in highly reduced Cd bioaccessibility. The presence of bacteria (food source) stimulates *C. elegans* pharyngeal pumping, which promotes the uptake of dissolved and bacterial-bound Cd^19^.

### Comparing OCR and growth inhibition

Similar ECx values for OCR and growth inhibition of *C. elegans* were evidenced (Table 1). Furthermore, the correlations between OCR and growth inhibition for BAC-C16 and Cd were strong [R^2^ = 0.93 (*P* = 0.001) and R^2^ = 0.91 (*P* = 0.001), respectively] and thus support the relationship evidenced between nematode length and OCR in the food density bioassay.

Lastly, this protocol and the use of *C. elegans* as a test organism can be further studied by, for example, investigating the sensitivity of *C. elegans* organismal vs. cellular respiration^15–17^. Considering that for the tested toxicants, OCR was equally sensitive to growth (an accepted and routinely used endpoint of toxicity^1,29^) we have clearly demonstrated that OCR inhibition can be used as an alternative method that facilitate high-throughput and rapid measurements.

### Quality control

A number of steps were taken to maximise the confidence in the generated results. Firstly, for accurate OCR measurements, oxygen consumption from other organisms than the test organism should be excluded. Since *E. coli*, serving as food source for the *C. elegans*, is an aerobic bacterium, the bacterial cultures had to be inactivated before use [see Stock preparation of the food source (*Escherichia coli*)]. Additionally, Penicillin-Streptomycin (Pen-Strep) was added to each well (see Experimental procedure) to ensure the inactivation of the *E. coli* food source and prevent bacterial contamination. Bacterial inactivation was checked by measuring the OCR of four extra wells with inactivated bacteria, but without nematodes (see Bioassay plate layout). In case of any oxygen consumption in these “E. coli” controls, the whole bioassay plate was discarded.

Secondly, temperature drifts occur during the operation of the Seahorse respirometer (see Temperature requirements). However, using a Seahorse respirometer, Koopman *et al*.^16^ studied the OCR of L4 and adult *C. elegans* and found no significant (*P* > 0.05) difference in OCR between 20 °C and 25 °C. Also in this study, OCR data measured with the Seahorse respirometer at 20 °C and 24.5 °C were not significantly different (*P* > 0.05), indicating that temperature shifts in this range have only a negligible influence on the outcome of the bioassays.

Lastly, all wells of the bioassay plate should be sufficiently oxygenated during OCR measurement in order to avoid inaccurate readings. During this study, the unprocessed oxygen level data (in mmHg) was checked (following OCR measurement) in order to confirm that anoxic conditions were never reached.

### Final considerations

The results presented in this study provide support for the use of *C. elegans* OCR inhibition as a functional and ecologically relevant endpoint of toxicity. Although different methods and instruments can be utilized, the high-throughput capability of modern respirometers facilitate simultaneous and rapid toxicity measurement of substances at various concentrations. Also, with further advancement and optimization of respirometers (e.g. better temperature management) it is likely that preparation and operating times will be reduced and measurement accuracy increased. Lastly, the authors recommend to explore more appropriate bacterial inactivation methods (e.g. without the addition of chemicals), or even the use of anaerobic bacteria as a food source.

## Methods

### Cultures and reagents

Cultures of *C. elegans* N2 and *E. coli* OP50 (food source) were obtained from the Caenorhabditis Genetics Centre (https://cbs.umn.edu/cgc/home). Stock solutions of sterile M9 medium (buffer) and cholesterol were prepared, as well as cultures of *C. elegans* reared, following ISO10872^29^. Penicillin-Streptomycin (Gibco 100X) was obtained from Thermo Fisher Scientific while the remaining reagents used in this study were obtained from Sigma-Aldrich^®^.

### Stock preparation of the food source (*Escherichia coli*)

An important step in the preparation of food stocks is the culturing, inactivation, and density adjustment of *E. coli* before the commencement of a bioassay. *Escherichia coli* was cultured, washed, and pelleted according to ISO10872^29^. The pellet was re-suspended in M9 medium after which an aliquot was diluted (1 → 10) and the optical density (OD) measured at 595 nm^35^ using a Pharo 300 Spectroquant (spectrophotometer). Finally, the density of the *E. coli* suspension was adjusted to an OD of 3 (595 nm). Thereafter, 5 mL aliquots of food stocks were transferred to 15 mL conical centrifuge tubes and heat inactivated (30 min at 65 °C) using a water bath^36^. The stocks were stored at −80 °C for a maximum of two weeks.

### Synchronization of *Caenorhabditis elegans*

**Figure 7:**
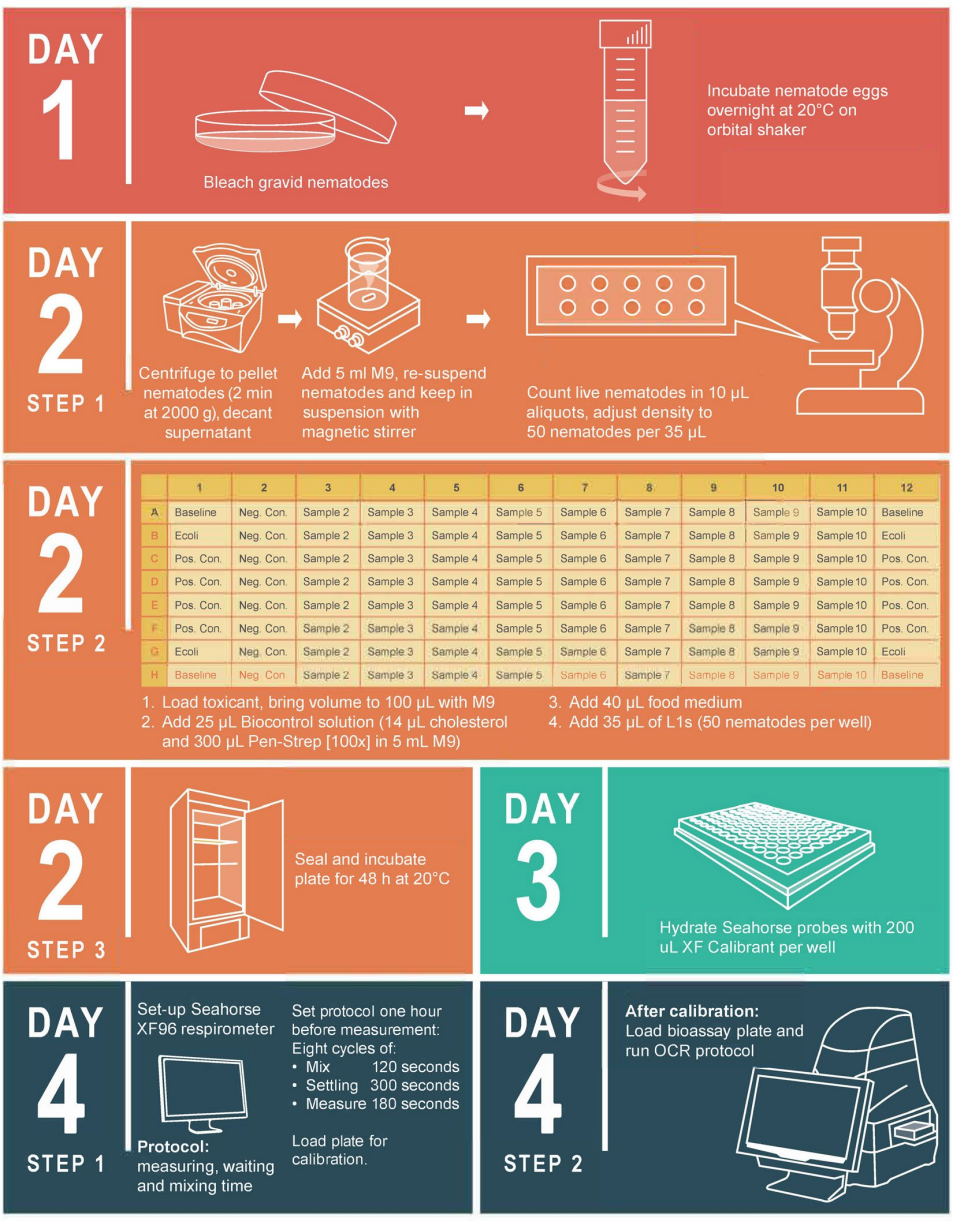
Experimental setup. Schematic overview of the experimental procedure and associated timeline for culturing *Caenorhabditis elegans* and its bacterial food source (*Escherichia coli* OP 50). The timeline for preparing and incubating the bioassay plate, as well as for *C. elegans* oxygen consumption rate (OCR) measurement using the Seahorse XF^e^96 Extracellular Flux Analyzer, is also provided.

A schematic overview of the experimental procedures, as well as the appropriate timeline, is provided in Fig. 7. On day one, *C. elegans* eggs were extracted from culture plates using the sodium hypochlorite (bleaching) method, followed by three wash cycles to remove any residual chemicals^15^. Subsequently, synchronized larval stage one (L1) nematodes were obtained after overnight (12–20 h) incubation in 50 mL sterile conical centrifuge tubes at 20 °C on an orbital shaker (100 rpm)^15^. This procedure also kills and dissolves *E. coli*, which could otherwise influence the outcome of the bioassay. It should be noted that prior to bleaching, the culture plates were studied using a Nikon SMZ1000 stereo microscope (100x magnification) to confirm the presence of eggs and gravid females.

### Number of nematodes

An important consideration is the number of nematodes per well to be used for OCR measurements. In order to effect a broad OCR range, 50 L1s per well were used. Koopman *et al*.^16^ reported this to be the largest number of larval stage four (L4) (expected life stage after incubation) nematodes to be used per well without inducing anoxic conditions.

### Food density and nematode development bioassay

Since food availability can have a substantial impact on *C. elegans* development^36^, a food density (0.1–0.7 OD, 595 nm) bioassay (see Bioassay plate layout) was performed (as described in Experimental procedure), with 50 L1s per well. The OCR of *C. elegans* was measured as detailed in Seahorse respirometer setup and oxygen consumption rate measurement.

### Toxicant stock solutions

In order to test the viability of OCR inhibition of *C. elegans* as a sublethal endpoint of toxicity, benzylcetyldimethylammonium chloride monohydrate (BAC-C16) and cadmium (Cd) were selected as toxicants. Benzylcetyldimethylammonium chloride monohydrate is routinely used as a positive control for *C. elegans* growth inhibition^27–29^, while Cd is regarded as an environmentally relevant, non-essential metal^19,37,38^.

The exposure solutions for BAC-C16 (made up in M9 medium) had the following concentrations: 2, 4, 6, 8, 10, 12, 16, 20, and 24 mg/L. For Cd, the exposure concentrations in M9 medium were 0.25, 0.5, 0.75, 1, 0.25, 1.5, 1.75, and 2 mg/L. For both assays (designed, performed, and measured as described in the following sections) the negative control consisted of M9 medium.

### Bioassay plate layout

The bioassay was carried out in 96-well culture plates, which will from hereon be referred to as ‘bioassay plates’. The bioassay plate layout (Fig. 7) was designed to allow for the maximum number of exposure concentrations with sufficient replication. The four corners (A1, H1, A12, and H12) represented the baseline wells, which the Seahorse respirometer uses for background correction of zero oxygen consumption. Four wells (B1, G1, B12, and G12) labelled “Ecoli” were reserved for *E. coli* food stocks (0.6 OD, 595 nm) containing a biocontrol solution (Pen-Strep and cholesterol), brought to a final volume of 200 µL with M9 medium. These wells were included to ensure that zero *E. coli* oxygen consumption, which could substantially impact OCR measurements, occurred (also see Quality Control).

Wells C1 – F1 and C12 – F12 were assigned for future use as a positive control for which BAC-C16 is recommended at a concentration of 8.94 mg/L (EC50 value of OCR inhibition as determined during this study). For the positive control the oxygen consumption rate inhibition of *C. elegans* (compared to the negative control), should range between 20% and 80%^29^. Column 2 was represented by the negative control, while columns 3 to 11 were used for the measurement of nine treatments with eight replicates each.

### Experimental procedure

Working in a sterile environment, the following preparation steps were executed on day two (Fig. 7):

1. A tube of *E. coli* food stock was allowed to thaw and reach room temperature.
2. Synchronized L1 nematodes were pelleted (2 min at 2000x g) and the supernatant discarded in order to remove residual material. Next, the pellet was re-suspended in 5 mL M9 medium using a magnetic stirrer. It should be noted that studying L1 nematodes using a Nikon Eclipse 50i light microscope (1000x magnification) after being subjected to stirring revealed no physical damage. Similarly, Van Aardt *et al*.^39^ reported that stirring speed had no effect on the OCR of *Meloidogyne incognita* (Kofoid & White, 1919) Chitwood, 1949 second-stage juveniles.
3. Suspended nematodes were transferred, in 10 µL aliquots, to a microscope slide and counted using a Nikon SMZ1000 stereo microscope (40-100x magnification). The average number of nematodes in 10 replicates were calculated per 1 µL. Thereafter, the concentration of nematodes in suspension was adjusted to 50 individuals per 35 µL.
4. Stock solutions of the studied toxicants (BAC-C16 and Cd) were prepared at twice the concentration of the highest exposure concentration as stock solutions were diluted 1:2 with food, biocontrol solution (see below), and nematode suspensions (Fig. 7). By taking into account the final volume (200 µL) of each well, the required volume of toxicant stock was calculated for each exposure concentration using the following equation:
  $${V}_{1}=\frac{{C}_{2}{V}_{2}}{{C}_{1}}$$
  where *V*_1_ represents the volume (unit: µL) of toxicant stock, *C*_2_ the final exposure concentration (unit: mg/L), *V*_2_ the final well volume of 200 µL, and *C*_1_ the toxicant stock concentration (unit: mg/L).
5. Biocontrol solution: A solution of cholesterol stock (14 µL), Pen-Strep (200 µL), and M9 medium (5 mL) was prepared. Cholesterol is necessary for the development of *C. elegans*^40^, while Pen-Strep ensured the inactivation of the *E. coli* food source and prevented bacterial contamination^35^. Pen-Strep is frequently used in the cultivation of *C. elegans*^41,42^ and was not expected to significantly influence the results. It is also important to consider that Pen-Strep were added to both control and exposure wells, which therefore renders it potential effect (if any) on the final results negligible.
  Immediately following these preparation steps, the bioassay plate (Fig. 7) was loaded in the following sequence:
6. The required volume of M9 medium was added to ensure a final volume per well of 200 µL.
7. The calculated volume of toxicant stock per exposure concentration was loaded.
8. *E. coli* food stocks were briefly vortexed at room temperature after which 40 µL was added to all bioassay wells (excluding ‘Baseline’ wells). This ensured a final OD of 0.6 (595 nm).
9. Finally, 25 µL of the biocontrol solution were added to all bioassay wells (excluding ‘Ecoli’ wells).
10. The bioassay plate was placed on an orbital shaker (100 rpm) for 15 min in order to ensure sufficient mixture of reagents.
11. Lastly, 35 µL of the nematode solution was added to each control and exposure well. No nematodes were added to the ‘baseline’ or ‘Ecoli’ wells. Using a Nikon TS100 inverted microscope (40-100x magnification) each well was checked to ensure correct loading of reagents and nematodes.

The bioassay plate was sealed with parafilm and incubated for 48 h at 20 °C. This incubation period was chosen to effect high OCR readings without risk of oxygen depletion during measurement^16^.

Additional considerations for the execution of this protocol follows:

- A final volume of 200 µL was assayed in order to simplify the calculation of the concentration and volume of solutions. However, according to the manufacturer (Agilent Technologies, Santa Clara, United States) the final well volume can range between 150 µL and 275 µL^43^. Therefore, the protocol can be adjusted accordingly if lower or higher well volumes are required.

### Seahorse respirometer setup and oxygen consumption rate measurement

#### Cartridge hydration

The Seahorse respirometer makes use of optic fibre bundles, which insert into solid state sensor probes (containing polymer embedded fluorophores) and emit light to excite the fluorophores. These optic fibres then measure the change in fluorophore emission resulting from the change in oxygen concentration and thus serves as an indirect measurement of oxygen consumption. On day three (Fig. 7), the Seahorse respirometer cartridge, which houses the probes, was hydrated by adding 200 µL XF calibrant to each well followed by overnight incubation at 37 °C.

#### Temperature requirements

The Seahorse respirometer was designed for cell OCR measurement at 37 °C, contrary to *C. elegans’* typical culture temperature range of between 16 and 25 °C^18^. While the instrument is capable of regulating the temperature in this range, it requires a room temperature of 4 °C, which was logistically not possible. Therefore, the room was cooled to the lowest possible temperature of 16 °C. The respirometer’s temperature was set to 24 °C and the internal heater switched off. It should be noted that the Seahorse respirometer generates heat during operation and was therefore only powered on directly before use. On day four (Fig. 7), the Seahorse cartridge was removed from the incubator two hours prior to use and left to cool and reach ambient temperature (16 °C).

#### Seahorse settings

Prior to OCR measurement, the following Seahorse respirometer protocol was programmed (Fig. 7):

1. **Two min mixing**: This involves the raising and lowering of the cartridge in order to replenish the oxygen levels within each well.
2. **Five min waiting**: The cartridge remains stationary in the ‘raised’ position to allow the nematodes to settle.
3. **Three min measuring**: The cartridge is lowered and a microchamber (of 3 µL) is created at the bottom of each well in which nematode oxygen consumption is measured. The decrease in oxygen is converted to a single OCR value per well.

This represents one measurement cycle, which was repeated eight times. After programming and 60 min prior to the OCR test, the Seahorse XF cartridge was inserted into the Seahorse respirometer for calibration. The calibration step is standard in every Seahorse analysis and cannot be unselected. During this calibration of the sensors, the instrument reads the coefficients of the sensor cartridge and bioassay plate to ensure accurate data acquisition. Once calibrated, the bioassay plate containing the nematodes was inserted into the Seahorse respirometer after which it underwent an equilibration period (12 min) during which temperature stability across the plate is ensured, followed by the above detailed OCR protocol. Oxygen levels during and after OCR measurement were checked to ensure that anoxic conditions were not induced. The Agilent Seahorse Wave 2.4 software package was used for exporting OCR data.

Upon completion of OCR measurement, 100 µL Bengal Rose (used here as a nematode staining agent) was added to each well and the bioassay plate heat inactivated (10 min at 80 °C)^29^. Bioassay plates were stored, for a maximum of 7 days, at 4 °C. Nematode length was measured and growth calculated as described in ISO10872^29^.

### Statistical analyses

#### Food density bioassay

The average OCR (of the eight measurement cycles) and length of *C. elegans* were calculated per well and graphically illustrated, at different food densities, using GraphPad Prism 7 software package. Thereafter, the density of food required to allow maximum nematode development was calculated using a segmented regression model. Briefly, the growing phase of the curve was fitted with a quadratic model. The plateau, in turn, was fitted using a constant representing a line running parallel to the density food axis defining the maximum nematode development. The plateau point was determined under a condition of continuity and smoothness as defined in the supplementary material^44^. This analysis was performed using SAS/STAT software package 9.4.

The relationship between OCR and nematode length was explained by an exponential growth (non-linear) regression model. In order to further study this relationship, the dependant variable (OCR) was log_10_ transformed and a linear regression model fitted. The 95% confidence limits were also calculated. These graphs were created and analyses performed using GraphPad Prism 7 software package.

#### Toxicant concentration-response bioassays

The average nematode OCR and growth of the eight measurement cycles were calculated per exposure concentration for the BAC-C16 and Cd concentration-response bioassays. Using ToxRat Professional 3 software package, the percentage decrease per exposure concentration was calculated against the negative control. Thereafter, the Probit analysis using the linear maximum likelihood regression algorithm was performed. The Chi-squared test was used to indicate the goodness-of-fit of the regression line. Furthermore, the effective concentrations (EC10, EC20, and EC50) for OCR and growth inhibition of *C. elegans* were calculated, while 95% confidence limits were based on Fieller’s Theorem. Lastly, in order to study the relationship between OCR and growth inhibition of *C. elegans*, the data were tested for normality using the D’Agostino & Pearson omnibus normality test^45^. The data presented a normal distribution and therefore the Pearson correlation coefficient test was performed.

#### Oxygen consumption rate response to temperature fluctuations

In order to determine whether temperature had a significant effect on the OCR of *C. elegans*, the negative control data (of eight measurements) of the two toxicant bioassays were used. Temperatures during both assays ranged between 20 °C and 24.5 °C. Firstly, the bioassay data were tested for normality using the D’Agostino & Pearson omnibus normality test^45^. Thereafter, the significance between the measurement means were tested using a one-way analysis of variance (ANOVA) (parametric data) or Kruskal-Wallis test (non-parametric data) with Tukey’s test and Dunn’s test applied, respectively, for multiple comparisons. These analyses were performed using GraphPad Prism 7 software package.

## Supplementary information

Supplementary material.

## Data Availability

Data will be made available by the corresponding author upon request.
