# Supplementary material for: Oxygen consumption rate of Caenorhabditis elegans as a high-throughput endpoint of toxicity testing using the Seahorse XFe96 Extracellular Flux Analyzer
Source: Sci Rep. 2020 Mar 6;10:4239. doi: 10.1038/s41598-020-61054-7 (PMC7060326; doi:10.1038/s41598-020-61054-7)
Supplement: Supplementary file 1 — Supplementary material. [file 41598_2020_61054_MOESM1_ESM.pdf]

Oxygen consumption rate of *Caenorhabditis elegans* as a high-throughput endpoint of toxicity testing using the Seahorse XF^{e}96 Extracellular Flux Analyzer

G. Du Preez[1,2,*], H. Fourie[1], M. Daneel[2], H. Miller[3], S. Höss[4,5], C. Ricci[6,7], G. Engelbrecht[1], M. Zouhar[1,8] & V. Wepener[1]

[1]Unit for Environmental Sciences and Management, North-West University, Private Bag X6001, Potchefstroom, 2520, South Africa

[2]Agricultural Research Council – Institute for Tropical and Subtropical Crops, Private Bag X11208, Nelspruit, 1200, South Africa

[3]Human Metabolomics, Faculty of Natural Sciences, North-West University, Potchefstroom, 2520, South Africa

[4]Ecossa, Giselastrasse 6, 82319 Starnberg, Germany

[5]University of Bielefeld, Department of Animal Ecology, Konsequenz 45, 33615 Bielefeld, Germany

[6]Centre of Excellence for Nutrition (CEN), North-West University, Potchefstroom, South Africa

[7]Pediatric Epidemiology, Department of Pediatrics, University Medicine Leipzig, Leipzig, Germany

[8]Czech University of Life Sciences, Faculty of Agrobiology, Food and Natural Resources, Department of Plant Protection, Kamycka 129, 165 21 Prague, Czech Republic

*Corresponding author: gerhard.dupreez@nwu.ac.za

Segmented model

The segmented model states that for any value of x less than x_0 the expected value of Y is a quadratic function, while for values of x greater than x_0 the mean of Y is the constant c.

$$E[Y|x] = \begin{cases} \alpha + \beta x + \gamma x^2 & if\ x < x_0 \\ c & if\ x \geq x_0 \end{cases}$$

Continuity and smoothness conditions were imposed to the two segments of the model. Firstly, the continuity condition was obtained so that the quadratic and the plateau section meet at x_0. Secondly, the first derivative with respect to x was set to 0 at x0.

Continuity condition

$$E[Y|x_0] = \alpha + \beta x_0 + \gamma x_0^2$$

Smoothness condition

$$\frac{\delta E[Y|x_0]}{\delta x} = \beta x_0 + 2\gamma x_0 \equiv 0$$

Solving the equation for x_0 and substitute into the expression for c, the two conditions are jointly satisfied when:

$$x_0 = - {}^{-\beta}/_{2\gamma}$$

$$c = \alpha - {}^{\beta^2}/_{4\gamma}$$

Please see SAS (2013) for additional information.

SAS, 2013. SAS/STAT User's Guide. SAS Institute, Cary.
